# Tumor-targeted dual-action NSAID-platinum(iv) anticancer prodrugs†

**DOI:** 10.1039/d3qi00968h

**Published:** 2023-06-28

**Authors:** Alexander Kastner, Theresa Mendrina, Florian Bachmann, Walter Berger, Bernhard K. Keppler, Petra Heffeter, Christian R. Kowol

**Affiliations:** a University of Vienna, Faculty of Chemistry, Institute of Inorganic Chemistry Waehringer Str. 42 1090 Vienna Austria christian.kowol@univie.ac.at; b Center of Cancer Research and Comprehensive Cancer Center, Medical University of Vienna Borschkegasse 8a 1090 Vienna Austria petra.heffeter@meduniwien.ac.at; c Research Cluster “Translational Cancer Therapy Research” 1090 Vienna Austria; d University of Vienna, Vienna Doctoral School in Chemistry (DoSChem) Waehringer Str. 42 1090 Vienna Austria

## Abstract

Platinum(iv) prodrugs are a promising class of anticancer agents designed to overcome the limitations of conventional platinum(ii) therapeutics. In this work, we present oxaliplatin(iv)-based complexes, which upon reduction, release acetylsalicylic acid (aspirin), known for its antitumor activity against colon cancer and currently investigated in combination with oxaliplatin in a phase III clinical study. Comparison with a recently reported cisplatin analog (asplatin) revealed a massive increase in reduction stability for the oxaliplatin complex in mouse serum. This was in line with the cell culture data indicating the desired prodrug properties for the newly synthesized complex. For *in vivo* studies, a new derivative containing an albumin-binding maleimide unit was synthesized. Indeed, distinctly longer plasma half-life as well as higher tumor accumulation in comparison to asplatin and oxaliplatin were observed, also leading to significantly higher antitumor activity and overall survival of CT26 tumor-bearing mice.

## Abstract 2

We investigated the first oxaliplatin(iv) complexes releasing acetylsalicylic acid (aspirin) upon reduction. The albumin-targeted derivative showed distinctly improved antitumor activity compared to asplatin, a recently reported cisplatin(iv) analog.

## Graphical abstract

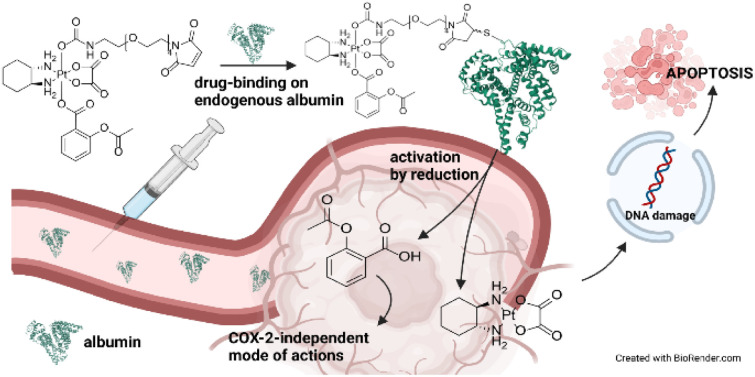

## Introduction

Cancer remains one of the leading causes of death, with nearly 10 million cases worldwide in 2020.^1^ Among the treatment strategies employed, the use of platinum(ii) complexes has been prominent since the discovery of cisplatin in 1965.^2,3^ Over the following decades, two additional platinum(ii) complexes, namely carboplatin and oxaliplatin, received worldwide approval. Additionally, selected Asian countries have approved neda-, hepta-, loba-, dicyclo-, and miriplatin for clinical use.^4^ However, despite their effectiveness, these platinum(ii) complexes are associated with various dose-limiting side effects and the problem of drug resistance.^5^ One way to overcome these drawbacks is the development of inert platinum(iv) counterparts, which are activated more selectively once they reach the tumor tissue.^6,7^ Furthermore, they allow for additional functionalization of the axial ligands. This can be utilized for tuning physicochemical properties, the attachment of various bioactive ligands or tumor-specific drug delivery.^7–9^

**Fig. 1:**
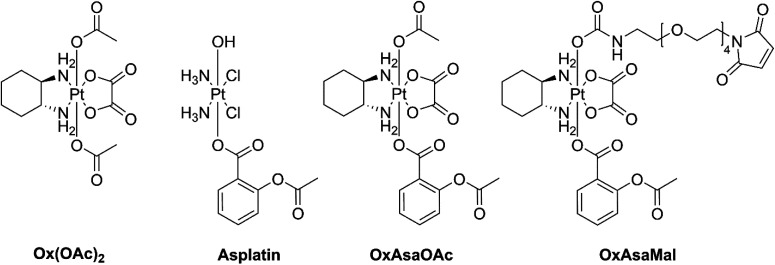
Overview of the investigated compounds in this study.

Cyclooxygenase-2 (COX-2) is an enzyme responsible for catalyzing the biosynthesis of prostaglandins, which have several protumorigenic effects.^10^ Many cancer types highly express COX-2 and prostaglandin E2 as response to the typical chronic inflammation.^11^ Non-steroidal anti-inflammatory drugs (NSAIDs) are well-known COX-1/2 inhibitors and are currently in clinical trials as anticancer agents.^12,13^ The NSAID acetylsalicylic acid (aspirin, Asa) is also a promising candidate for adjuvant cancer therapy.^14^ The activity of Asa originates, on the one hand, from the aforementioned COX-2 inhibition; on the other hand, there are also non-COX-dependent pathways *e.g. via* transcription factor nuclear factor κb (NFκb), AMPK/mTor or TGF-β1-dependent downregulation of Bcl-2.^15,16^ Furthermore, Asa influences the tumor microenvironment *e.g.* by downregulation of the proinflammatory cytokine interleukin-17 in CD4^+^ T-cells as well as decrease of tumor infiltration with immunosuppressive regulatory T-cells (T_regs_).^17^ Of note, based on their exciting synergism, Asa is currently being tested in combination with oxaliplatin against colorectal cancer in a phase III clinical trial (study number: JCOG1503C).^18^ Therefore, it is a highly interesting and elegant strategy to combine these two drugs into one chemical entity, *e.g.* by exploiting platinum(iv) chemistry. For example, Liu and coworkers showed that Asa-conjugated cisplatin(iv) (asplatin; Fig. 1) exerted distinctly improved activity and was able to overcome cisplatin drug resistance.^19^ This compound was recently also enclosed in calix[*n*]arenes which resulted in superior anticancer activity and cancer selectivity *in vitro*.^20^ Moreover, Hey-Hawkins and coworkers attached indomethacin and ibuprofen to *cis*- and oxaliplatin(iv), which resulted in enhanced activities as well as reduced side-effects by decreasing tumor-associated inflammation.^21,22^ More recently, Aldrich-Wright and coworkers attached indomethacin and Asa onto platinum(iv) complexes with polyaromatic equatorial ligands, which also showed higher activity than their parental complexes.^23^ Interestingly, despite the aforementioned phase III clinical trial in colorectal cancer, so far no oxaliplatin(iv) complexes with Asa have been synthesized and investigated. Consequently, in the here presented study, we report on the synthesis of novel oxaliplatin(iv) complexes bearing Asa as additional bioactive moiety (Fig. 1). Subsequently, biological tests and uptake studies were conducted in cell culture in order to investigate the impact on cancer cells in comparison to asplatin. Additionally, for improved tumor targeting a maleimide-bearing albumin-binding derivative was synthesized. Finally, the tissue distribution and anticancer activity of the new maleimide-Asa-platinum(iv) complex was investigated in a murine cancer model *in vivo*.

## Results and discussion

### Synthesis, stability and reduction behavior

OxAsaOAc (Fig. 1) was synthesized in 42% yield from OxOAcOH, *via* coupling of the symmetric anhydride of **Asa** (Scheme S1†) and purified *via* preparative HPLC. Furthermore, asplatin and Ox(OAc)_2_ were prepared according to literature as reference compounds.^24^ All complexes were characterized in detail by NMR spectroscopy, mass spectrometry and elemental analysis.

**Fig. 2:**
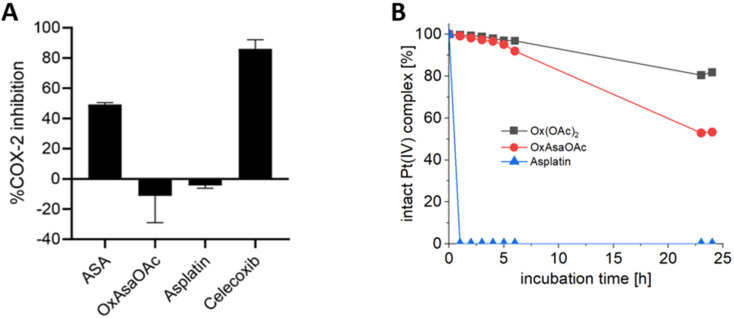
(A) Inhibition of human-recombinant COX-2. Asa, asplatin and OxAsaOAc were used at a final concentration of 250 μM. Celecoxib was used at 10 μM. (B) Reduction kinetics of 1 mM OxAsaOAc, Ox(OAc)_2_ and asplatin in 250 mM phosphate buffer (pH = 7.4) at 20 °C with 10 eq. of ascorbic acid over 25 h, measured with UHPLC.

To investigate the different properties of OxAsaOAc, Ox(OAc)_2_ and asplatin, we first analyzed the stability in aqueous solution *via* HPLC measurements. 1 mM of each compound were incubated in 250 mM phosphate buffer (pH 7.4) at 20 °C for 25 h (Fig. S1†) revealing high stability for all complexes. The prodrug nature of our compounds could be further proven by cell-free COX-2 inhibition assays, where; in contrast to free Asa and the specific COX-2 inhibitor Celecoxib, no impact on the enzyme was observed (Fig. 2A). Next, the reduction kinetics of the platinum complexes at 1 mM in 250 mM phosphate buffer (pH 7.4) at 20 °C with the addition of 10 eq. ascorbic acid was investigated over 24 h (Fig. 2B). Asplatin was immediately reduced and already at the second time point (1 h), no remaining intact platinum(iv) complex could be observed. This fits to previously published data of comparable cisplatin(iv) analogues.^25^ In contrast, OxAsaOAc and Ox(OAc)_2_ were highly stable, with 92% and 97% intact complex after 6 h, respectively. After 24 h, 81% of intact Ox(OAc)_2_ remained, while 53% were left for OxAsaOAc. The slower reduction kinetics of Ox(OAc)_2_ compared to the Asa-bearing complex originates most probably from the electron-withdrawing effect of the benzoic acid moiety. In addition, also the release of Asa and oxaliplatin (for OxAsaOAc and Ox(OAc)_2_) could be detected confirming the observed differences (Fig. S2;† in case of asplatin the slow decrease of released Asa most probably originates from hydrolysis of Asa; *vide infra*).

**Fig. 3:**
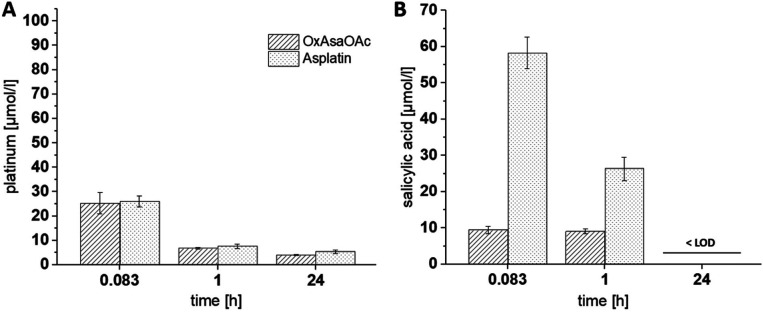
(A) Platinum levels in serum collected from mice 10 min, 1 h and 24 h after a single i.v. administration of asplatin and OxAsaOAc at doses equimolar to 9 mg kg^−1^ oxaliplatin, measured with ICP-MS. (B) Levels of released salicylic acid in these samples, after extraction with acetonitrile (salicylic acid levels after 24 h were below the LOD of 3 μM), measured with UHPLC-MS.

Next, we were interested, whether this behavior also impacts on the Asa release from the compounds *in vivo*. To allow these evaluations, as a first step, a protocol of Grobecker *et al.* with addition of HCl/H_3_PO_4_ and extraction of Asa with MeCN (see experimental part) was established.^26^ To this end, we spiked Asa in fresh murine blood, isolated the serum, applied the extraction protocol and measured the samples *via* HPLC-MS. It is well known that Asa hydrolyzes in aqueous solutions to salicylic acid which occurs even faster in blood.^27^ Accordingly, already after the 30 min necessary for blood clotting and serum extraction, more than half of Asa was hydrolyzed to salicylic acid (Fig. S3†). Having this protocol established, we treated healthy tumor-free Balb/c mice once with equimolar concentrations of asplatin or OxAsaOAc, and blood was drawn 10 min, 1 h and 24 h after treatment. ICP-MS measurements confirmed that the platinum levels between the two drugs were very similar at all three time points (Fig. 3A). In contrast, in the HPLC-MS measurements of the same samples, strong differences in the released Asa levels could be observed (Asa could only be detected as salicylic acid) (Fig. 3B). In more detail, asplatin showed the highest amount of free salicylic acid present at 10 min (∼30 mol% of the administered asplatin dose) and 1 h. In comparison, OxAsaOAc showed distinctly lower levels of free salicylic acid (<5 mol% of administered dose) at both time points. No salicylic acid could be detected in any of the 24 h samples (>LOD of 3 μM). These results are in accordance to our cell-free reduction experiments, indicating low stability for asplatin.

### Anticancer activity in cell culture

To evaluate whether the observed differences in the reduction kinetics influence the biological activities of the compounds, the cytotoxicity of oxaliplatin and its prodrugs Ox(OAc)_2_ and OxAsaOAc as well as cisplatin and its derivative asplatin was determined in cell culture after 72 h treatment. Since oxaliplatin is clinically used against gastrointestinal cancers, MTT assays were performed on a colorectal cancer cell line panel. With respect to the free platinum(ii) species, the cells were more sensitive to oxaliplatin than cisplatin (Table 1), which is in good agreement to the fact that oxaliplatin is approved for therapy of this tumor entity.^28^ Thus, in case of RKO and HCT116, IC_50_ values even in the nanomolar range were observed. Cisplatin showed a 2- to 8-fold lower anticancer activity. HT29 cells were the most resistant against both platinum(ii) drugs.

**Table 1:** Anticancer activity was determined by MTT assays after 72 h treatment. IC_50_ values (μM) are given as mean ± SD. COX-2 expression was characterized by Western Blot and RT-PCR

Cell line	IC50 ± SD (μM)						COX-2 levels
	Oxaliplatin	Ox(OAc)_2_	OxAsaOAc	Cisplatin	Asplatin	Celecoxib	
Caco-2	1.4 ± 0.2	34.1 ± 1.9	17.9 ± 2.1	4.5 ± 1.0	6.4 ± 1.1	79.2 ± 3.6	−
RKO	0.4 ± 0.04	9.0 ± 1.0	4.3 ± 0.4	1.6 ± 0.3	0.9 ± 0.0	79.3 ± 4.9	−
SW480	1.0 ± 0.2	13.2 ± 2.5	11.2 ± 1.3	7.8 ± 1.3	0.6 ± 0.1	83.1 ± 1.5	−
HT-29	5.3 ± 1.2	135.9 ± 34.6	32.7 ± 5.5	10.1 ± 0.3	9.2 ± 0.6	68.0 ± 6.4	++
HCT116	0.8 ± 0.1	17.4 ± 2.4	9.9 ± 1.5	3.1 ± 0.5	2.2 ± 0.1	76.2 ± 4.3	−
HCT116/OxR	27.8 ± 5.2	>100	42.8 ± 4.3	10.4 ± 1.8	8.7 ± 1.8	79.2 ± 2.7	−
CT26 (murine)	1.8 ± 0.2	22.5 ± 2.8	12.8 ± 0.8	1.2 ± 0.2	2.5 ± 0.2	60.9 ± 2.5	++

With regard to the oxaliplatin-releasing platinum(iv) complexes Ox(OAc)_2_ and OxAsaOAc, both drugs had higher IC_50_ values than the free platinum(ii) species, which is in accordance with the expected prodrug nature and slow reduction of these drugs.^29,30^ However, in most cell lines, OxAsaOAc was about 2-fold more active than Ox(OAc)_2_. The only exceptions were SW480 cells with similar activity and HT29, where an even 4-fold higher activity of OxAsaOAc was observed. In contrast, asplatin was similar (Caco-2, HT-29, HCT116) or even more active (RKO and SW480) than cisplatin in our cell line panel, questioning the prodrug nature of this platinum(iv) complex. Noteworthy, with respect to the impact of oxaliplatin resistance of HCT116/OxR cells, OxAsaOAc was distinctly less affected than oxaliplatin with a resistance factor of **4.3***vs.***34.1**, while no significant difference was observed for asplatin*vs.*cisplatin. COX-2 is physiologically induced in response to pro-inflammatory stimuli and growth factors upon inflammatory processes.^31^ In addition, also cancer cells can have enhanced COX-2 expression. In order to assess the role of COX-2 in the observed *in vitro* anticancer activities of the drugs, COX-2 levels of the tested cell line panel were assessed by western blotting and PCR (Fig. S4†). In addition, the sensitivity of the panel against the very specific COX-2 inhibitor celecoxib was tested. It was not possible to test Asa alone due the strong acidification of the medium by the free acid. These investigations revealed, on the one hand, that HT-29 and the murine CT26 cells were the only COX-2-positive models in our panel, and, on the other hand, that COX-2 inhibition by celecoxib had only limited effect on the viability of the cancer cells in general (Table 1). Thus, in case of the COX-positive cells, only a non-significant trend towards reduced IC_50_ values in comparison to the other cells was observed. Consequently, this indicates that the increased activity of asplatin compared to cisplatin in some cell lines is not based on COX-2 inhibition but other mechanisms. Only the enhanced efficiency of OxAsaOAc*vs.*Ox(OAc)_2_ in HT-29 cells (compared to the other Cox-2-negative cell models) could be associated with COX-2 inhibition by the intracellularly released Asa. In general, these experiments are in good agreement with a recent study by Ravera *et al.*,^32^ who compared asplatin with cisplatin-releasing platinum(iv) prodrugs carrying the COX-2 inhibitors ketoprofen and naproxen in axial position, respectively. In this study the enhanced anticancer activity of the new drugs was attributed to increased cellular drug uptake due to higher lipophilicity and, to some extent, activation of NAG-1, an anti-tumorigenic and pro-apoptotic protein.

### Impact of the Asa ligand on the cellular drug uptake

**Fig. 4:**
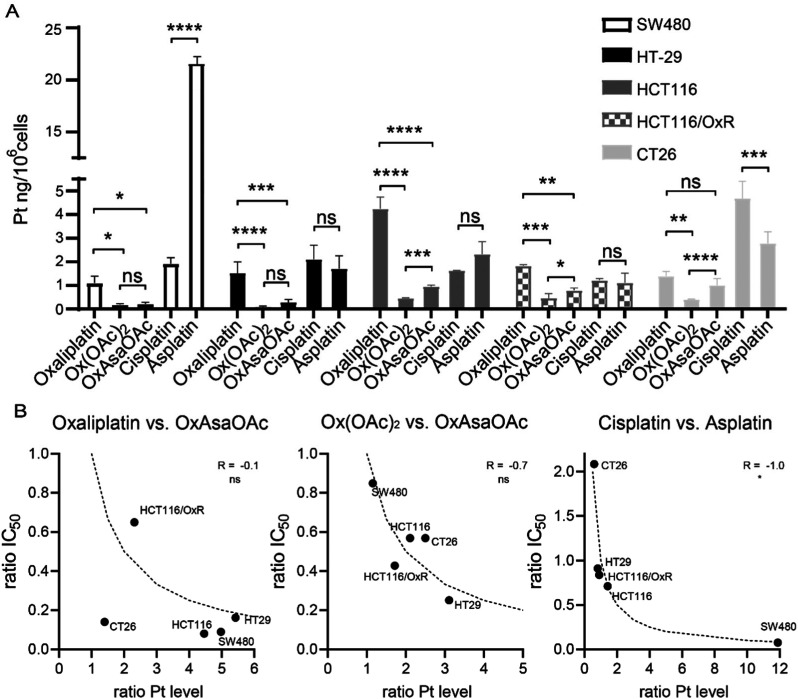
Cellular platinum levels after 2 h incubation with 10 μM of the indicated compounds measured by ICP-MS. (A) Bars indicate mean ± SD of triplicates. Significance was calculated by two-way ANOVA and Tukey's multiple comparisons test (ns – non significant, *p < 0.05, **p < 0.01, ***p < 0.001, ****p < 0.0001). (B) Correlation of intracellular platinum levels and IC_50_ values. Ratio of platinum content and IC_50_ values of OxAsaOAc*vs.*oxaliplatin or Ox(OAc)_2_ and asplatin*vs.*cisplatin were calculated and correlated in GraphPad Prism using two-tailed Spearman correlation (ns – non significant, *p < 0.05). Dotted lines indicate the calculated correlation curve of −1.0.

To check whether the difference in IC_50_ values can be explained by different drug uptake, intracellular platinum levels were measured by ICP-MS after treatment (Fig. 4A). In more detail, 5 cell lines of our panel were treated for 2 h and total lysates were collected by digestion with concentrated HNO_3_. In general, cisplatin and oxaliplatin had a similar drug uptake pattern with exception of HCT116 which showed preferential oxaliplatin uptake, and CT26, which had higher cisplatin accumulation. In good agreement with previous reports,^30^ HCT116/OxR had reduced oxaliplatin levels compared to the parental model, which confirms impaired drug uptake as an important factor in the resistance of these cells. In addition, we were able to repeat previous observations that Ox(OAc)_2_ is characterized by a distinctly decreased cellular uptake compared to oxaliplatin.^29^ However, its intracellular drug levels were not affected by the resistance of HCT116/OxR cells. This suggests a different route of drug uptake between the platinum(ii) and platinum(iv) drugs. With regard to the impact of the Asa ligand, in most of the tested cell lines, OxAsaOAc treatment led to ∼2-fold higher intracellular platinum levels than Ox(OAc)_2_. In contrast, for cisplatin and asplatin the pattern was not so clear: in SW480 cells, a distinctly pronounced asplatin uptake was observed, while the uptake of the two drugs was rather similar in HT-29 cells and the two HCT116 clones. In CT26, cisplatin treatment led to higher intracellular platinum levels than asplatin treatment. Considering that also the sensitivities of the cell lines against the drugs varied, we evaluated whether there is a correlation (by Spearman correlation) between the ratios of the IC_50_ values and the ratios of the measured intracellular platinum content (Fig. 4B). Noteworthy, there was no correlation between OxAsaOAc and oxaliplatin (*R* = −0.1), and also in case of OxAsaOAc and Ox(OAc)_2_, only a non-significant trend with *R* = −0.7 was observed. In contrast, there was a very strong and significant correlation in case of asplatin compared to cisplatin (*R* = −1.0, *p* < 0.05). This further supports the hypothesis that, especially in case of asplatin, the COX-2 inhibition does not play a role in the activity against the cancer cells *in vitro*, but the differences in activity are mainly based on the intracellular platinum content.

### Anticancer activity of oral doses of OxAsaOAc and asplatin

It is well known that COX-2 is involved in the paracrine signaling between immune cells, resulting in a pro-tumorigenic tumor microenvironment.^10,33^ Consequently, we got interested in the impact of the Asa ligand on the anticancer activity of the drugs *in vivo*. Therefore, we chose immune-competent murine colon cancer CT26 allografts as test system, which were characterized by high COX-2 expression (Fig. S4†). Moreover, a pilot experiment indicated responsiveness of this tumor model to both oxaliplatin (9 mg kg^−1^ i.v.) as well as Asa (50 mg kg^−1^ p.o., Fig. S5†). Noteworthy, this experiment also revealed that the reported MTD of 9 mg kg^−1^ for oxaliplatin already resulted in toxic side effects of the drug in female Balb/c mice. Thus, the experiment was stopped on day 19 due to a distinct drop in body weight in these animals. As Asa is clinically applied p.o., we tested the efficacy of OxAsaOAc and asplatin in an oral application setting. Preliminary tolerability experiments in tumor-free mice confirmed that OxAsaOAc at 52.8 mg kg^−1^ (a concentration equivalent to 15 mg kg^−1^Asa and 33 mg kg^−1^oxaliplatin) was well tolerable. However, in case of asplatin, only a concentration of 20.7 mg kg^−1^ (equivalent to 7.5 mg kg^−1^Asa and 12.5 mg kg^−1^ cisplatin) could be used. This reduced tolerability could probably be explained by the lower stability of asplatin and, consequently, higher toxicity of released cisplatin. For the therapy experiment, the animals were treated with OxAsaOAc and asplatin for five subsequent days over two weeks. Disappointingly, in these settings none of the drugs had any impact on tumor growth (Fig. S6†). In contrast to its activity at 50 mg kg^−1^, also Asa had no impact on tumor growth at the used dose of 15 mg kg^−1^. Consequently, we concluded that the drug doses reaching the tumor in these therapy settings were insufficient, which prompted us to aim for carrier systems with enhanced tumor-targeting properties.

### Generation of albumin-targeted OxAsa derivatives

**Fig. 5:**
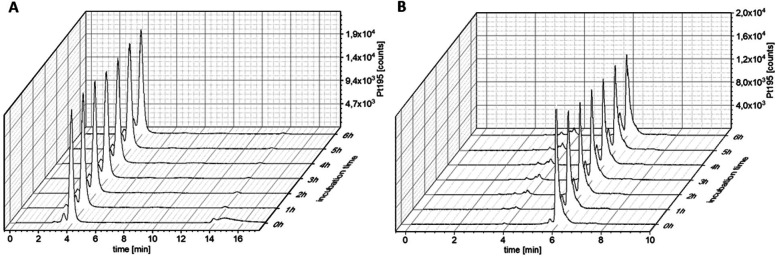
^195^Pt-traces of incubation of 100 μM (A) OxAsaMal and (B) OxAsaOAc in FCS (containing 150 mM phosphate buffer, pH = 7.4) at 37 °C over 6 h, measured with SEC-ICP-MS.

One type of nanotransporter, which can be used elegantly for tumor targeting, is coupling to human serum albumin. For this approach, maleimide chemistry can be exploited, which results in endogenous conjugation of the drug to the single free thiol group Cys34 in the blood stream.^29,30,34^ In turn, this results not only in increased plasma half-life time of drugs but also in enhanced drug accumulation *via* the enhanced permeability and retention (EPR) effect.^9^ Consequently, as a next step, we replaced the OAc in OxAsaOAc by a maleimide moiety (OxAsaMal) (Fig. 1). With regard to the synthesis, Asa was first coupled to Ox(OH)_2_, again using the anhydride to yield the asymmetric complex OxAsaOH (Scheme S1†). The maleimide, containing a PEG-4 linker in order to increase solubility, was coupled to OxAsaOH after conversion of the carboxylic acid to an isocyanate to yield OxAsaMal. Comparable to OxAsaOAc (compare Fig. S1†), the incubation in phosphate-buffered saline (PBS) at pH 7.4 showed high stability of the platinum core over 24 h, and in addition the well-known hydrolysis of the maleimide moiety at physiological pH (data not shown).^35^ With regard to the reduction kinetics in the presence of ascorbic acid, the direct measurement of OxAsaMal was prevented due to maleimide hydrolysis. However, as mentioned above, the released Asa and oxaliplatin could be monitored (Fig. S2†), which confirmed a slow reduction behavior of OxAsaMal comparable to OxAsaOAc (compare Fig. 2). The ability of OxAsaMal to bind to albumin was analyzed *via* size exclusion chromatography coupled to inductively coupled plasma mass spectrometry (SEC-ICP-MS) with OxAsaOAc as reference. The complexes were dissolved in fetal calf serum (FCS, buffered with 150 mM phosphate buffer to ensure a stable pH), incubated at 37 °C (Fig. 5), and their ^195^Pt and ^48^SO content were measured. The sulfur trace shows the eluting proteins, including albumin (Fig. S7†), while the platinum trace indicates whether the complex is protein-bound (eluting at the same time as the albumin fraction, 2–4 min) or still in its free form (eluting in the low molecular weight fraction (LMWF), >5 min) (Fig. 5). Already at the 0 h time point, a large fraction of OxAsaMal was bound (77%) to the albumin fraction (Fig. 5A), increasing to 95% after 1 h of incubation. The remaining peak is most likely the hydrolyzed maleimide complex, unable to bind to albumin anymore. The non-maleimide reference OxAsaOAc, on the other hand, did not bind to albumin (or any other protein) to a significant degree (<5%) and thus was visible in the LMWF even after longer incubation time of up to 6 h (Fig. 5B). The growing peak at ∼5.8 min most probably originates from the hydrolysis of the Asa ester moiety in the OxAsaOAc complex (see below).

### Impact of albumin binding on the serum levels and organ distribution of the Asa-releasing prodrug

**Fig. 6:**
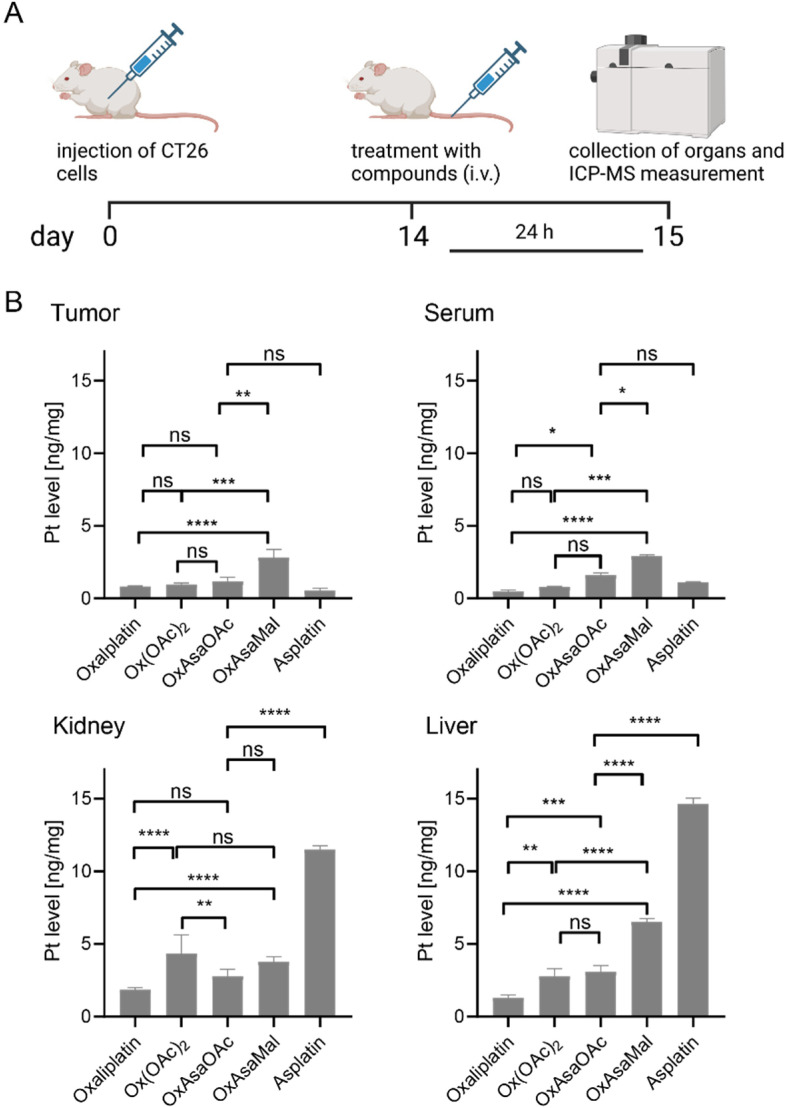
Plasma levels and drug distribution *in vivo*. (A) Experimental overview. CT26-bearing female Balb/C mice were treated once *via* the tail vein with the indicated drugs at doses equimolar to 9 mg kg^−1^ oxaliplatin. After 24 h, animals were sacrificed, and serum, tumor tissue, and diverse organs were collected. (B) Platinum levels in isolated tissues were detected by ICP–MS and normalized to tissue weight. Oxaliplatin data were taken as a reference from our previous study.^36^ Significance was calculated by two-way ANOVA and Tukey's multiple comparisons test (ns – non significant, *p < 0.05, **p < 0.01, ***p < 0.001, ****p < 0.0001).

As a next step, we tested the impact of the maleimide-mediated albumin binding on the serum levels and organ distribution *in vivo* using CT26 tumor-bearing Balb/c mice. Briefly, the mice were treated once *via* tail vein injection with OxAsaOAc, asplatin or OxAsaMal at doses equimolar to 9 mg kg^−1^ oxaliplatin. In addition, Ox(OAc)_2_ was included as a reference (oxaliplatin data were used from our previous study).^36^ After 24 h, animals were anesthetized and blood collected by heart puncture. The animals were sacrificed, and tumor as well as liver and kidneys collected. Platinum levels were assessed by ICP-MS from HNO_3_-digested samples. Indeed, the albumin binding resulted in significantly higher plasma levels and, in turn, higher tumor accumulation of OxAsaMal compared to all four non-albumin-binding drugs (Fig. 6). Noteworthy, the liver and kidney samples of the asplatin-treated animals were exceptionally high, which would be in line with the observed reduced tolerability of asplatin compared to the oxaliplatin derivatives observed in the tolerability experiments above.

### Anticancer activity of OxAsaMal*in vivo*

**Fig. 7:**
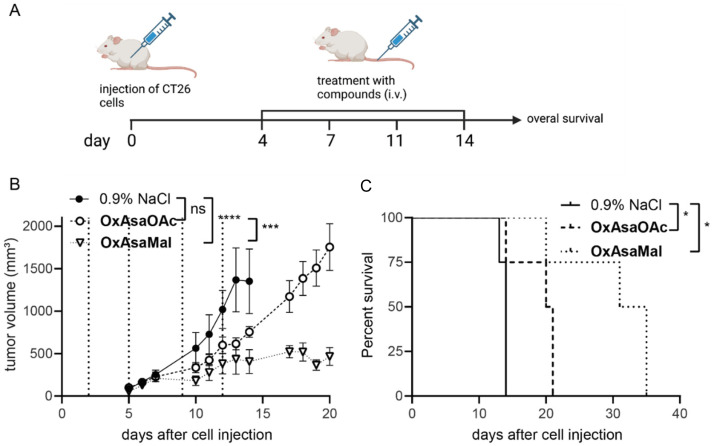
Anticancer activity against CT26 allografts in immune-competent Balb/c mice. (A) Schematic timeline of the performed therapy experiment in CT26-bearing Balb/c mice. (B) Impact on tumor growth. Data are presented as means ± SEM. Dashed lines indicate treatments of solvent, OxAsaOAc, OxAsaMal (i.v.) equimolar to 9 mg kg^−1^ oxaliplatin. Significance was calculated using two-way ANOVA and Tukey's multiple comparison test (ns-non significant, ***p < 0.001, ****p < 0.0001). (C) Overall survival is depicted *via* Kaplan–Meier curve. Statistical significance was calculated using long-rank test and Mantel-Cox posttest (*p < 0.05).

Finally, we were interested, whether the albumin binding of OxAsaMal resulted in enhanced anticancer activity compared to OxAsaOAc. Thus, CT26 tumor-bearing Balb/c mice were treated with the drugs 4-times over 2 weeks with drug doses equimolar to 9 mg kg^−1^ oxaliplatin, and the impact on tumor volume was monitored by regular caliper measurements (Fig. 7A). The compounds were applied i.v., as this is essential for the albumin-targeting delivery strategy, and the prior p.o. treatment attempts did not show any activities. Both drugs were well tolerated at these conditions (Fig. S8†). Interestingly, although 3.5-fold lower drug doses were applied compared to the p.o. experiments described above, OxAsaOAc had significant impact on both tumor growth as well as overall survival (Fig. 7B). This indicates an insufficient bioavailability of OxAsaOAc upon oral application. The activity could be further improved by introduction of the albumin-binding maleimide group. In more detail, treatment with OxAsaMal led to a long-lasting disease control over several days and, consequently, ∼20 days prolonged overall survival of the animals.

## Conclusion

In this study, we synthesized oxaliplatin-based platinum(iv) complexes with an axial Asa ligand (OxAsaOAc and OxAsaMal). Of note, both compounds showed distinctly slower reduction rates and higher overall serum stability *in vivo* in comparison to the recently reported cisplatin analogue asplatin. Consequently, the oxaliplatin-based compounds indeed revealed the desired prodrug nature of platinum(iv) complexes. This also indicates that, in general, for a suitable prodrug design, the selection of the platinum(ii) core is a crucial parameter. Cisplatin(iv) derivatives widely lack the required stability of a prodrug, but sometimes show higher cell uptake and activity than their platinum(ii) counterparts due to increased lipophilicity. However, especially for drug delivery systems, this persistence is important, as they should be stably bound to their carrier over a long time while circulating in the bloodstream. Such highly stable platinum(iv) complexes can only be achieved with *e.g.* oxaliplatin (or carboplatin) derivatives. This could impressively be shown for OxAsaMal, exhibiting long plasma half-life and tumor accumulation *in vivo*. Finally, this also resulted in significantly less tumor growth and prolonged survival compared to oxaliplatin. Overall, this study shows that the selection of the platinum core can dramatically influence stability, pharmacokinetics and activity profile of platinum(iv) prodrugs and that the conjugation of oxaliplatin(iv) and Asa as a bioactive ligand is a promising strategy.

## Materials and methods

### Chemicals and instrumentation

Potassium tetrachloridoplatinate (K_2_[PtCl_4_]) was purchased from Johnson Matthey (Switzerland). Water for synthesis was taken from a reverse osmosis system. For HPLC measurements Milli-Q water (18.2 MΩ cm, Merck Milli-Q Advantage, Darmstadt, Germany) was used. Other chemicals and solvents were purchased from commercial suppliers (Sigma Aldrich, Merck and Fisher Scientific). Electrospray ionization (ESI) mass spectra were recorded on a Bruker Amazon SL ion trap mass spectrometer in positive and/or negative mode by direct infusion. High resolution mass spectra were measured on a Bruker maXis™ UHR ESI time of flight mass spectrometer. One- and two-dimensional ^1^H-NMR and ^13^C-NMR spectra were recorded on a Bruker Avance III 500 or AV III 600 spectrometer at 298 K. For ^1^H-NMR spectra the solvent residual peak was taken as internal reference. Elemental analysis measurements were performed on a PerkinElmer 2400 CHN Elemental Analyzer at the Microanalytical Laboratory of the University of Vienna. The compounds were purified by preparative RP-HPLC using a Waters XBridge C18 column on an Agilent 1200 Series system. Milli-Q water and acetonitrile were used as eluents with a flow rate of 17 ml min^−1^, unless otherwise stated.

### Synthesis of complexes

The platinum(iv) complexes Ox(OH)_2_,^37^OxOAcOH ^37^ and Ox(OAc)_2_ ^25^ as well as asplatin ^24^ and Asa anhydride^38^ were synthesized according to literature (Scheme S1†).

#### (OC-6-44)-(2-acetoxybenzoato)[(1*R*,2*R*)-1,2-cyclohexanediamino]hydroxidooxalatoplatinum(iv); OxAsaOH

Ox(OH)_2_ (800 mg, 0.58 mmol) was suspended in 4 ml abs. DMSO and stirred at 10 °C. Aspirin anhydride (620 mg, 0.70 mmol) was dissolved in 0.5 ml abs. DMSO and added slowly over the course of 8 h and then stirred overnight under Ar. The solvent was removed under reduced pressure, the residue was dissolved in MeOH and precipitated with Et_2_O, filtered and dried *in vacuo*. Yield: 950 mg (86%) as a white-brown solid. The compound was used in the next step without further purification. ^1^H-NMR (500 MHz, DMSO-*d*_6_): *δ* = 8.41 (bs, 1H, N*H*_2_), 8.22 (bs, 1H, N*H*_2_), 7.86 (d, *J* = 7.7 Hz, 2H, C*H*_phe_), 7.61–7.55 (m, 2H, N*H*_2_), 7.52 (dd, *J* = 6.0 Hz, 1H, C*H*_phe_), 7.42 (dd, *J* = 7.6 Hz, 2H, C*H*_phe_), 7.23 (bs, 1H, N*H*_2_), 2.64 (bs, 2H, C*H*_cHex_), 2.14–2.03 (m, 2H, C*H*_2,cHex_), 1.58–1.38 (m, 4H, C*H*_2,cHex_), 1.19–1.03 (m, 2H, C*H*_2,cHex_) ppm.

#### 1-(14-isocyanato-3,6,9,12-tetraoxatetradecyl)-1*H*-pyrrole-2,5-dione; Mal-PEG4-NCO

1-(2,5-dioxo-2,5-dihydro-1*H*-pyrrol-1-yl)-3,6,9,12-tetraoxapentadecan-15-oic acid (100 mg, 0.288 mmol) was dissolved in 3 ml acetone and 3 ml abs. toluene. NEt_3_ (46 μl, 0.318 mmol, 1.1 eq.) and ethyl chloroformate (34 μl, 0.318 mmol, 1.1 eq.) were added while the solution was stirred on −5 °C. After 10 min stirring, NaN_3_ (32 mg, 0.288 mmol, 1 eq.) was added and the solution was stirred for 1.5 h at RT. Acetone and generated ethanol were removed under reduced pressure and the remaining toluene phase was dried with MgSO_4_ and filtered. The solution was stirred for 2 h at 140 °C under reflux and Ar. The solvent was removed under reduced pressure. The compound was used in the next step without further purification. Yield: 70 mg (88%) as a clear oil. ^1^H-NMR (500 MHz, DMSO-*d*_6_): *δ* = 7.04 (s, 2H, C*H*_mal_), 3.59–3.39 (m, 20H, C*H*_2_) ppm.

#### (OC-6-44)-acetato(2-acetoxybenzoato)[(1*R*,2*R*)-1,2-cyclohexanediamino]oxalatoplatinum(iv); OxAsaOAc

OxOAcOH (200 mg, 0.423 mmol) was suspended in 5 ml abs. DMF. Aspirin anhydride (289 mg, 0.845 mmol) was added and the suspension was stirred overnight at RT under Ar, after which the solvent was removed *in vacuo*. The crude product was purified by preparative RP-HPLC with an isocratic gradient of 20% acetonitrile. Yield: 112 mg (42%) as a white solid. ^1^H-NMR (500 MHz, DMSO-*d*_6_): *δ* = 8.52–8.47 (m, 1H, N*H*_2_), 8.43–8.38 (m, 1H, N*H*_2_), 8.34–8.28 (m, 1H, N*H*_2_), 8.26–8.21 (m, 1H, N*H*_2_), 7.77 (dd, *J* = 7.8, 1.7 Hz, 1H, C*H*_phe_), 7.56 (td, *J* = 7.5, 1.7 Hz, 1H, C*H*_phe_), 7.33 (td, *J* = 7.6, 1.2 Hz, 1H, C*H*_phe_), 7.12 (dd, *J* = 8.1, 1.0 Hz, 1H, C*H*_phe_), 2.65–2.57 (m, 2H, C*H*_cHex_), 2.20 (s, 3H, C*H*_3_), 2.14 (t, *J* = 12.4 Hz, 2H, C*H*_*2*,cHex_), 1.98 (s, 3H, C*H*_3_), 1.53 (d, *J* = 11.2 Hz, 2H, C*H*_*2*,cHex_), 1.48–1.42 (m, 2H, C*H*_*2*,cHex_), 1.22–1.08 (m, 2H, C*H*_*2*,c_Hex) ppm. ^13^C-NMR (DMSO-*d*_6_): *δ* = 178.4 (CH_3_*C*OOPt), 171.5 (C_phe_*C*OOPt), 169.2 (*C*O-CH_3_), 163.4 (*C*O_oxalate_), 163.3 (*C*O_oxalate_), 149.1 (*C*_phe_OAc), 132.7 (*C*H_phe_), 131.3 (*C*H_phe_), 125.8 (*C*_phe_COOPt), 125.7 (*C*H_phe_), 123.4 (*C*H_phe_), 61.1 (*C*H_cHex_), 60.9(*C*H_cHex_), 30.9 (*C*H_2,cHex_), 30.8 (*C*H_2,cHex_), 23.6 (*C*H_2,cHex_), 23.5 (*C*H_2,c_Hex), 22.9 (CH_3_), 20.9 (*C*H_3_) ppm; MS: calcd for [C_19_H_24_N_2_O_10_Pt-Na^+^]^+^ 658.10, found: 658.08; elemental analysis calcd for C_19_H_24_N_2_O_10_Pt·1.5 H_2_O: C: 34.43, H: 4.11, N: 4.23, found: C: 34.21, H: 3.79, N: 4.11.

#### (OC-6-34)-(2-acetoxybenzoato)[(1*R*,2*R*)-1,2-cyclohexanediamino]oxalato[(14-(2,5-dioxo-2,5-dihydro-1*H*-pyrrol-1-yl)-3,6,9,12-tetraoxatetradecyl)carbamato]platinum(iv); OxAsaMal

OxAsaOH (100 mg, 0.211 mmol) and Mal-PEG4-NCO (70 mg, 0.211 mmol) were dissolved in 3 ml abs. DMSO and stirred overnight at RT under Ar. The solvent was removed under reduced pressure and the crude product was purified using preparative HPLC with an isocratic gradient of 27% MeCN. Yield: 41 mg (24%) as a white solid. ^1^H-NMR (500 MHz, DMSO-*d*6): *δ* = 9.45 (bs, 1H, N*H*_2_), 8.75 (bs, 1H, N*H*_2_), 8.31 (bs, 2H, N*H*_2_), 8.22 (bs, 1H, N*H*_2_), 7.78 (dd, *J* = 7.4, 1.0 Hz, 1H, C*H*_phe_), 7.55 (td, *J* = 7.7, 1.0 Hz, 1H, C*H*_phe_), 7.33 (td, *J* = 7.7, 1.0 Hz, 1H, C*H*_phe_), 7.12 (dd, *J* = 8.1, 1.0 Hz, 1H, C*H*_phe_), 7.02 (s, 2H, C*H*_mal_), 6.80 (t, *J* = 5.4 Hz, 1H, C*H*_carbamate_), 3.57 (dd, *J* = 8.8, 3.1 Hz, 2H, NC*H*_2_), 3.53–3.43 (m, 16H, C*H*_2_), 3.11–3.01 (m, 2H, NHC*H*_2_), 2.65–2.55 (m, 2H, C*H*_cHex_), 2.20 (s, 3H, C*H*_3_), 2.17 (b, 2H, C*H*_2,cHex_), 1.56–1.50 (m, *J* = 6.6 Hz, 2H, C*H*_2,cHex_), 1.48–1.39 (m, 2H, C*H*_2,cHex_), 1.20–1.10 (m, 2H, C*H*_2,cHex_) ppm. ^13^C-NMR (DMSO-*d*_6_): *δ* = 171.5 (*C*OOPt), 170.9 (*C*O_mal_), 169.2 (*C*O–CH_3_) 164.2 (O*C*ONH_carbamate_), 163.4 (*C*O_oxalate_), 163.3 (*C*O_oxalate_), 149.2 (*C*_phe_OAc), 134.5 (*C*H_mal_), 132.7 (*C*H_phe_), 131.3 (*C*H_phe_), 125.8 (*C*H_phe_), 125.7 (*C*_phe_COOPt), 123.4 (*C*H_phe_), 69.8 (*C*H_2_), 69.7 (*C*H_2_), 69.6 (*C*H_2_), 69.5 (*C*H_2_), 69.4 (*C*H_2_), 69.2 (NHCH_2_*C*H_2_), 66.9 (NCH_2_*C*H_2_), 61.1 (*C*H_cHex_), 61.0 (*C*H_cHex_), 40.7 (NH*C*H_2_), 36.8 (N*C*H_2_), 31.0 (*C*H_2,cHex_), 30.9 (*C*H_2,cHex_), 23.6 (*C*H_2,cHex_), 23.5 (*C*H_2,cHex_), 20.9 (*C*H_3_) ppm; MS: calcd for [C_32_H_44_N_4_O_16_Pt-Na^+^]^+^ 958.23, found: 958.24; elemental analysis calcd for C_32_H_44_N_4_O_16_Pt·1.5 H_2_O: C: 39.93, H: 4.92, N: 5.82, found: C: 39.81, H: 4.72, N: 5.71.

### COX-2 inhibition assay

An enzyme immunoassay (EIA) kit (#560101, Cayman Chemicals) was performed to inhibit human recombinant COX-2. Assays were performed according to the manufacturer's protocol. Asa, asplatin and OxAsaOAc were used with a final concentration of 250 μM. Celecoxib was used with a final concentration of 10 μM.

Absorbance was measured at 410 nm with a Tecan Reader infinite® M200Pro (Tecan Group Ltd, Switzerland). COX-2 inhibition was calculated as instructed by the manufacturer protocol.

### Stability and reduction experiments

Phosphate buffer (250 mM, pH 7.4) containing 1 mM platinum compound was incubated at 20 °C, with and without the addition of 10 eq. l-ascorbic acid. The reaction was monitored on a Thermo Scientific Dionex UltiMate 3000 UHPLC-system using a Waters Acquity UPLC BEH C18 1.7 μm 3.0 × 50 mm column. Milli-Q water, containing 0.1% formic acid, and acetonitrile containing 0.1% formic acid were used as eluents. A gradient of 5–95% over 5 minutes was used. To evaluate the current state of the reaction, the peak area of the parental complex was used. This was done due to the fact, that in most cases the reduction products did not have a sufficient retention time to be distinguished from the injection peak.

### Extraction of released salicylic acid from serum

The extraction method for acetylsalicylic acid and salicylic acid from Grobecker and coworkers was used.^26^ For method validation and quantification, 99 μl mouse serum were spiked with Asa or salicylic acid dissolved in 1 μl DMSO in order to obtain a range of concentrations from 1 to 100 μM. Immediately afterwards the samples were acidified with 100 μl HCl/H_3_PO_4_ (0.1 M each) and extracted *via* addition of 600 μl of acetonitrile. After vigorous shaking for 1 min the suspension was stored at 4 °C for ten minutes before being centrifuged at 9500*g* for 10 min. The supernatant was taken up with a pipette and transferred into an Eppendorf tube with approx. 100 mg of solid NaCl. After vigorous shaking, the samples were again stored at 4 °C for 5 min and centrifuged at 9500 g for 5 min. The organic layer was taken up and measured on an Agilent 1260 Infinity II system using a Waters Acquity UPLC BEH C18 1.7 μm 3.0 × 50 mm column coupled to an Agilent InfinityLab LC/MSD mass spectrometer in negative mode. Milli-Q water, containing 0.1% formic acid, and acetonitrile containing 0.1% formic acid were used as eluents. A gradient of 5–95% acetonitrile in 6 min was used. The peak areas were determined and plotted for calibration (Fig. S9†).

### Cell culture

All cell cultures were grown at 37 °C in a humidified atmosphere containing 5% CO_2_. HCT116, RKO and HT-29 cells were cultured in McCoy's 5A Modified Media (Sigma-Aldrich, St. Louis, MO, USA) supplemented with 10% heat-inactivated fetal calf serum (FCS, PAA, Linz, Austria) and 2 mM glutamine (Sigma-Aldrich, St. Louis, MO, USA). The murine (Balb/c) colon cancer cell model CT26 (purchased from American Type Culture Collection, Manassas, VA, USA) were cultured in DMEM/F12 supplemented with 10% fetal calf serum (FCS). SW480 and Caco-2 cells were cultured in MEME media supplemented with 10% and 20% FCS, respectively. All cell culture media and reagents were purchased from Sigma-Aldrich Austria. The generation of the oxaliplatin-resistant HCT116 (HCT116/OxR) line by continuous exposure to oxaliplatin was previously published.^39^ The cells were selected every week with 10 μM oxaliplatin for 72 h. The cells were regularly checked for *Mycoplasma* contamination.

### Western blot analysis

Cells were seeded at a density of 4–7 × 10^6^ in 6-well plates in a total volume of 2 ml and allowed to recover overnight. Cells were harvested and proteins were isolated, resolved by sodium dodecyl sulfate-polyacrylamide gel electrophoresis and transferred onto a polyvinylidene difluoride membrane as previously described.^40^ The following primary antibodies were used: COX-2 (clone 33 (RUO), 1 : 250), 610204 BD transduction. The horseradish-peroxidase-conjugated anti-Mouse IgG secondary antibody (Fc-specific, A0168, 1 : 10000) was purchased from Merck KGaA (Darmstadt, Germany). Anti-β-actin (AC-15) (A5441, 1 : 2000) as a loading control was obtained from Sigma-Aldrich (St. Louis, MO, USA).

### RNA isolation and detection of COX-1/-2 levels by real-time PCR

Cells were seeded at a density of 4–7 × 10^6^ cells in 6-well plates in a total volume of 2 ml and incubated overnight. After incubation the medium was removed and total RNA was lysed and isolated from cells using the TRIzol reagent (Thermo Fisher Scientific, Massachusetts, USA), according to the manufacturer's instructions. RNA was transcribed to complementary DNA (cDNA) using RevertAid Reverse Transcriptase (EP0441, Thermo Fisher Scientific, Massachusetts, USA) and diluted 1 : 25. Real-time PCR was performed using Maxima SYBR Green/ROX qPCR Master Mix (2×) (K0221, Thermo Fisher Scientific, Massachusetts, USA) in a CFX96 Touch real-time PCR detection system (Bio-Rad Laboratories, California, USA). In order to detect the specific mRNA content the following primer sequences purchased from Eurofins Genomics were used: human β-actin: fwd (5′-GGA TGC AGA AGG AGA TCA CTG-3′), rev (5′-CGA TCC ACA CGG AGT ACT TG-3′); human PTGS1: fwd (5′-GGG GTT CTT ATT TTG CAT TCC-3′), rev (5′-ATT TGG GAT ACG AGC CAC TGT-3′); human PTGS2: fwd (5′-GAT CCC CAG GGC TCA AAC AT-3′), rev (5′-GAA AAG GCG CAG TTT ACG CT-3′); murine β-actin: fwd (5′-ATG GAG GGG AAT ACA GCC-3′), rev (5′-TTC TTT GCA GCT CCT TCG TT-3′); murine PTGS1 1: fwd (5′-GCC CTC TGT ACC CAA AGA C-3′), rev (5′-GGG CCA GAA GCT GAA CAT C-3′); murine PTGS2 1: fwd (5′-ACG CTT CTC CCT GAA GCC GTA C-3′), rev (5′-GTA GAG GGC TTT CAA TTC TGC AGC C-3′).

### Short-term anticancer activity assay (72 h)

Depending on their proliferation rate, cells were seeded at a density of 3500–5000 cells per well in 96-well microtiter plates and allowed to adhere overnight. Cells were exposed in triplicates to oxaliplatin, Ox(OAc)_2_, OxAsaOAc, cisplatin, asplatin and celecoxib in the indicated concentrations for 72 h. Cell viability was determined using the 3-(4,5-dimethylthiazol-2-yl)-2,5-diphenyltetrazolium bromide (MTT) assay (EZ4U, Biomedia, Vienna, Austria) according to the manufacturer's recommendation. Depending on the metabolic capacity, the cell lines were incubated between one and three hours at 37 °C and subsequently absorbance was measured at 450 nm (620 nm as a reference) with a Tecan Reader infinite® M200Pro (Tecan Group Ltd, Switzerland). Data were analyzed using Graph Pad prism (version 8.0.1) by using the point-to-point function. The curves allowed calculation of IC_50_ values by interpolation, as a parameter for cytotoxicity resulting in 50% reduction of cell viability compared to the untreated control cells. Following, dose–response curves were calculated using the point-to-point function.

### ICP-MS measurements

Cells were seeded at a density of 6 × 10^5^–8 × 10^5^ cells per well in respective cell culture medium in 6-well plates and left to recover overnight. Cells were treated in triplicates with 10 μM of the compounds for 2 h in parallel to three blank controls without cells. The supernatant was removed and all wells were carefully washed twice with PBS. The samples were digested using 500 μl 67% HNO_3_ for one hour at room temperature. 400 μl of each well were transferred into 7.6 ml H_2_O. Total platinum content was determined using a quadrupole-based inductively coupled plasma mass spectrometry (ICP-MS) instrument Agilent 7800 (Agilent Technologies, Tokyo, Japan) equipped with the Agilent SPS 4 autosampler (Agilent Technologies, Tokyo, Japan) and a MicroMist nebulizer at a sample uptake rate of approximately 0.2 ml min^−1^. The Agilent MassHunter software package (Workstation Software, Version C.01.04, 2018) was used for data evaluation. Additional two wells per condition were treated similar and used for determination of the total cell number. The blank values were subtracted and the platinum content per 10^6^ cells was calculated. The ICP-MS operation parameters are given in Table S1.†

### SEC-ICP-MS measurements

Fetal calf serum was purchased from Sigma-Aldrich and buffered with 150 mM phosphate pH 7.4 in order to guarantee a stable pH. The platinum(iv) complexes (5 mM) were dissolved in 150 mM phosphate buffer (pH 7.4) and diluted 1 : 50 in the buffered serum to obtain a final concentration of 100 μM. The samples were then incubated in the autosampler at 37 °C and analyzed every 1 h for 6 h. Between each sample a pure water blank was measured. For SEC-ICP-MS measurements an Agilent 1260 Infinity system coupled to an Agilent 7800 ICP-MS equipped with a dynamic reaction cell was used. Oxygen (purity 5.5, Messer Austria GmbH, Gumpoldskirchen, Austria) was used as reaction gas. HPLC parameters are given in Table S2† and ICP-MS operation parameters are given in Table S3.†

### Animals

All experiments were executed according to the regulation of the Ethics Committee for the Care and Use of Laboratory Animals at the Medical University Vienna. 8–12 weeks old BALB/c were kept in a pathogen-free environment with a 12 h light dark-cycle with *ad libitum* access to food and water. Every procedure was performed in a laminar airflow under sterile condition. Tumor growth and possible side effects of drug treatment were evaluated by daily recording the tumor size by caliper measurement according to the formula length × width^2^/2 and parameters of the animal's overall health conditions.

### Pharmacokinetic studies

Balb/c mice (female, *n* = 3 per treatment) were treated i.v. at concentrations equimolar to 9 mg kg^−1^ oxaliplatin in 0.9% NaCl (Ox(OAc)_2_ 12.1 mg kg^−1^, OxAsaOAc 15.0 mg kg^−1^, OxAsaMal 21.8 mg kg^−1^, asplatin 11.6 mg kg^−1^). After 10 min, 1 h and 24 h blood was collected *via* the facial vein. Serum was isolated by centrifugation at 900 g for 10 min for two times and stored at −80 °C. Platinum content was detected by ICP-MS measurements, using an Agilent 7800 (Agilent Technologies, Tokyo, Japan) equipped with an Agilent SPS 4 autosampler (Agilent Technologies, Tokyo, Japan) and a MicroMist nebulizer. The Agilent MassHunter software package (Workstation Software, Version C.01.04, 2018) was used for data evaluation. The ICP-MS operation parameters are given in Table S1.† Salicylic acid was extracted and detected as described above.

### Organ distribution in CT26-bearing BALB/c mice

CT26 cells (5 × 10^5^ cells in 50 μl serum-free medium) were injected subcutaneously (s.c.) into the right flank of female BALB/c mice. When tumors formed to a size of 500 mm^3^ the mice (*n* = 2 per group) were treated once with the compounds i.v. at concentrations equimolar to 9 mg kg^−1^ oxaliplatin in 0.9% NaCl (Ox(OAc)_2_ 12.1 mg kg^−1^, OxAsaOAc 14.1 mg kg^−1^, OxAsaMal 21.8 mg kg^−1^, asplatin 11.6 mg kg^−1^). After 24 h, the animals were anesthetized and blood was drawn. The animals were sacrificed by cervical dislocation and tumors as well as organs were collected for measurement with ICP-MS. To isolate serum and blood pellets, blood was centrifuged for 10 min at 3000 rpm. The supernatant characterized as serum was transferred to a new tube and centrifuged again to get rid of residual red blood cells.

### Anticancer activity in CT26-bearing BALB/c mice

CT26 cells (5 × 10^5^ cells in 50 μl serum-free medium) were injected s.c. into the right flank of female BALB/c mice. When the tumors were palpable, five groups were randomly formed: three control groups treated with oxaliplatin, Asa or solvent and three groups treated with OxAsaOAc, OxAsaMal and asplatin (*n* = 4 per group). OxAsaOAc, OxAsaMal and oxaliplatin were treated i.v. in concentrations equimolar to 9 mg kg^−1^ oxaliplatin in 0.9% NaCl. Due to high toxicity asplatin was treated i.v. in lower concentrations equimolar to 3 mg kg^−1^ cisplatin. Asa was treated orally using 50 mg kg^−1^ in 5% DMSO/0.9% NaCl. Every day, the animals were monitored for the overall health conditions. Additionally, tumor size was measured. In case of a decreased body weight of ∼20% or ulcerated tumors, animals were sacrificed by cervical dislocation and tumors as well as organs were collected.

## Conflicts of interest

There are no conflicts to declare.

## Supplementary Material

QI-010-D3QI00968H-s001
