# Supplementary material for: Tumor-targeted dual-action NSAID-platinum(iv) anticancer prodrugs
Source: Inorg Chem Front. 2023 Jun 28;10(14):4126–38. doi: 10.1039/d3qi00968h (PMC10334471; doi:10.1039/d3qi00968h)
Supplement: QI-010-D3QI00968H-s001 [file QI-010-D3QI00968H-s001.pdf]

Electronic Supplementary Material (ESI) for Inorganic Chemistry Frontiers.
This journal is © the Partner Organisations 2023

Supporting Information

Tumor-targeted dual-action NSAID-platinum(IV) anticancer prodrugs

Alexander Kastner[1,4‡], Theresa Mendrina[1,2,3‡], Florian Bachmann[1], Walter Berger[2,3], Bernhard K. Keppler[1,3], Petra Heffeter*[2,3] and Christian R. Kowol*[1,3]

[1] University of Vienna, Faculty of Chemistry, Institute of Inorganic Chemistry, Waehringer Str. 42, 1090 Vienna, Austria.

[2] Center of Cancer Research and Comprehensive Cancer Center, Medical University of Vienna, Borschkegasse 8a, 1090 Vienna, Austria.

[3] Research Cluster "Translational Cancer Therapy Research", 1090 Vienna, Austria.

[4] University of Vienna, Vienna Doctoral School in Chemistry (DoSChem), Waehringer Str. 42, 1090 Vienna, Austria.

E-Mail: christian.kowol@univie.ac.at, petra.heffeter@meduni.ac.at

‡These authors contributed equally to this work.

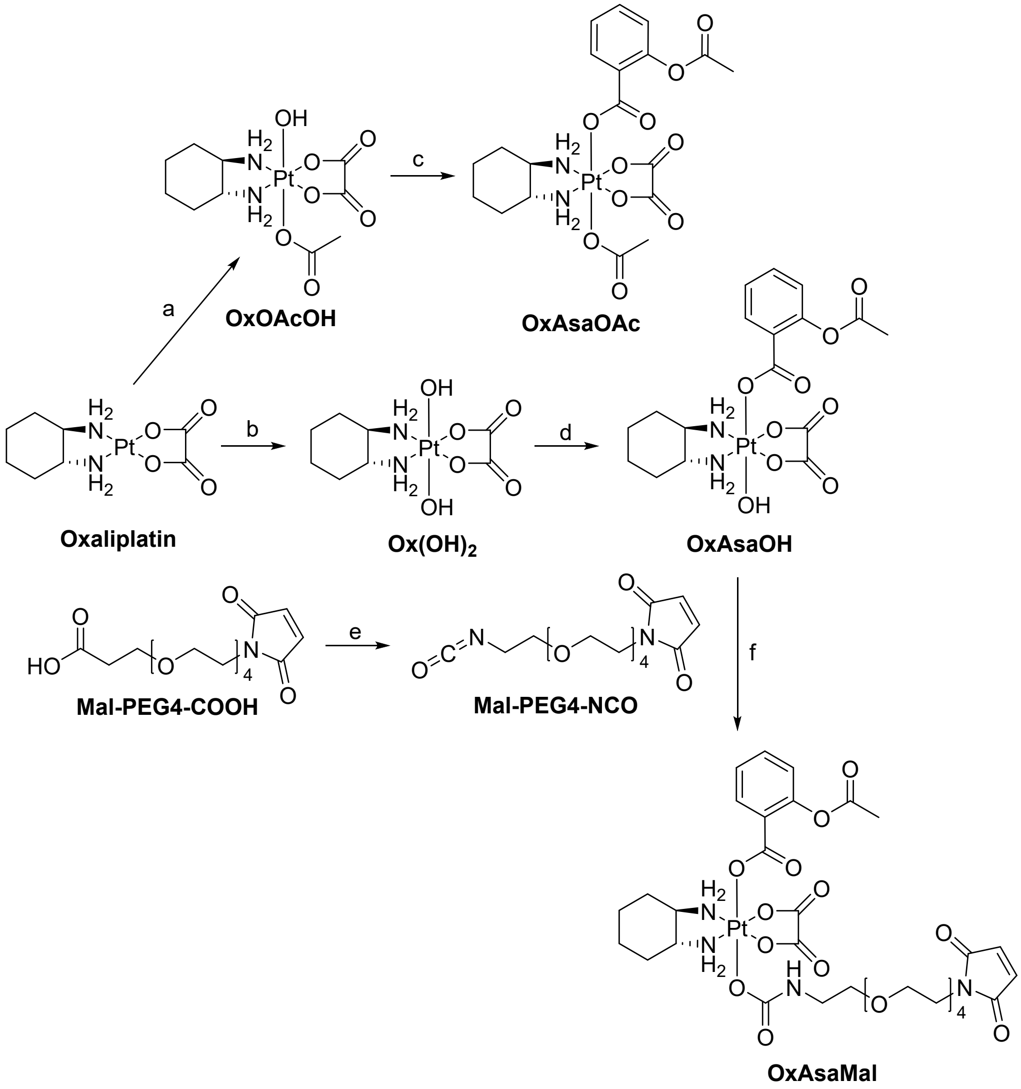

Scheme S1: Reaction overview of the synthesized complexes **OxAsaOAc** and **OxAsaMal**. a: H_2O_2 (50% w/w), AcOH, (45%); b: H_2O_2 (50% w/w), H_2O, (98%); c: aspirin anhydride, DMF, (42%); d: aspirin anhydride, DMSO, (86%); e: NEt_3, ethylchloroformate, NaN_3, acetone/toluene, (88%); f: Mal-PEG4-NCO, DMSO, (24%).

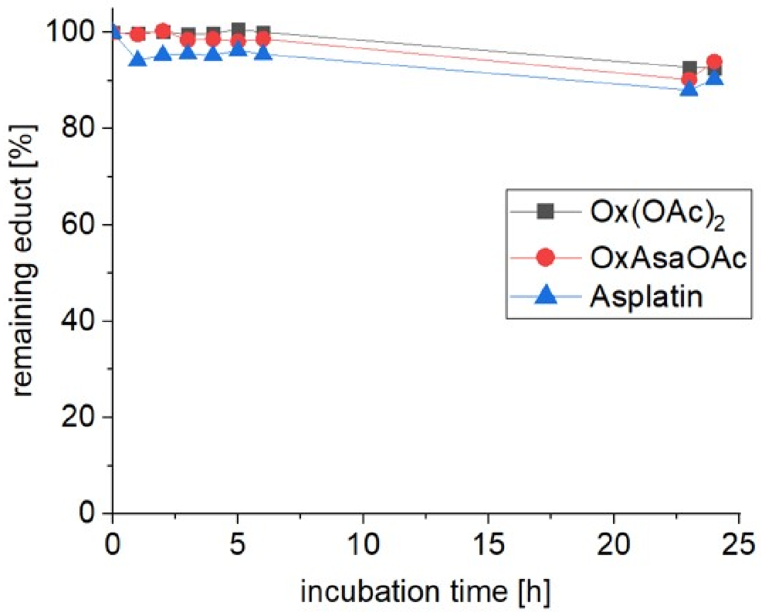

Figure S1: Stability of 1 mM **OxAsaOAc**, **Ox(OAc)$_2$** and **Asplatin** in 250 mM phosphate buffer (pH = 7.4) at 20°C over 25 h, measured with UHPLC.

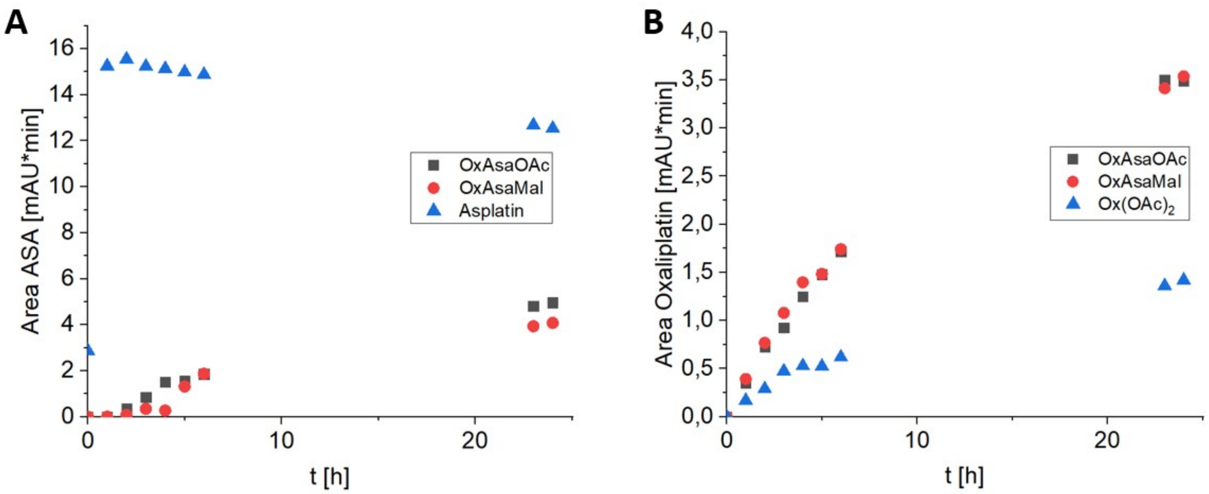

Figure S2: Reduction kinetics of 1 mM compound in 250 mM phosphate buffer (pH = 7.4) at 20°C with 10 eq. of ascorbic acid. Measured with HPLC and following (A) the released aspirin and (B) the released oxaliplatin.

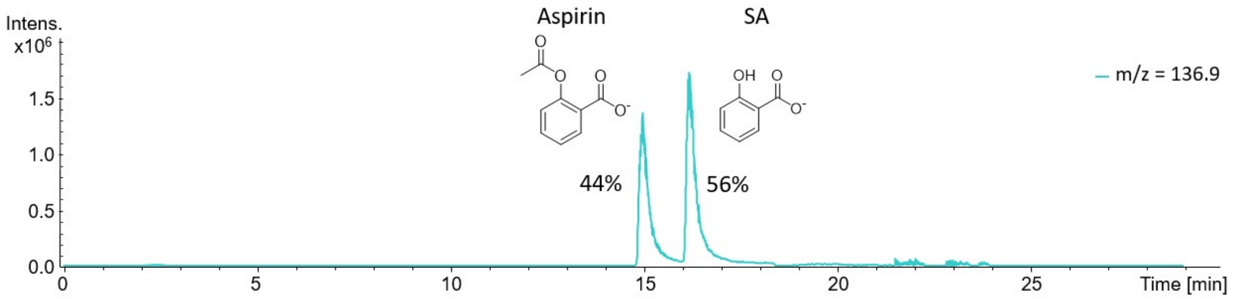

Figure S3: HPLC-MS trace of the extracted mouse serum showing the hydrolysis of aspirin. For aspirin (15 min) the same mass (m/z = 136.9) as for salicylic acid (SA, 16.2 min) was measured, as the acetate is cleaved during ionization.

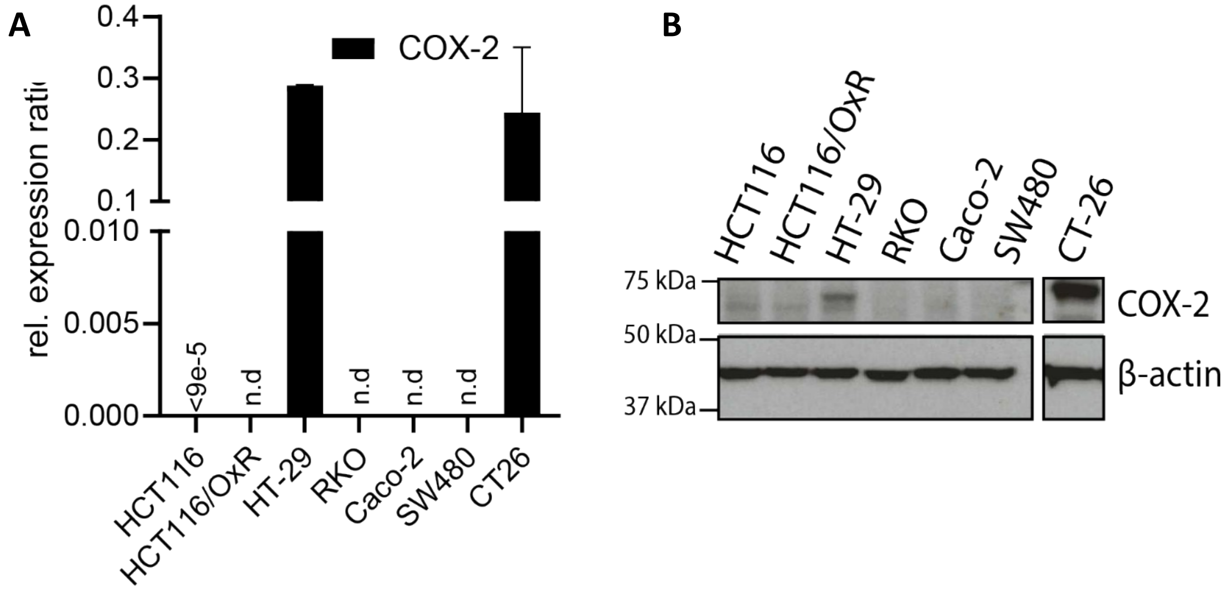

Figure S4: COX-2 expression in the cell lines from the test panel. (A) COX-2 mRNA levels of untreated cell lines. Quantification is described as a relative expression compared to ß-actin. (n.d = not detected). (B) COX-2 protein expression of untreated cell lines. COX-2 is detected at 72 kDa. As loading control, ß-actin (42 kDa) was used.

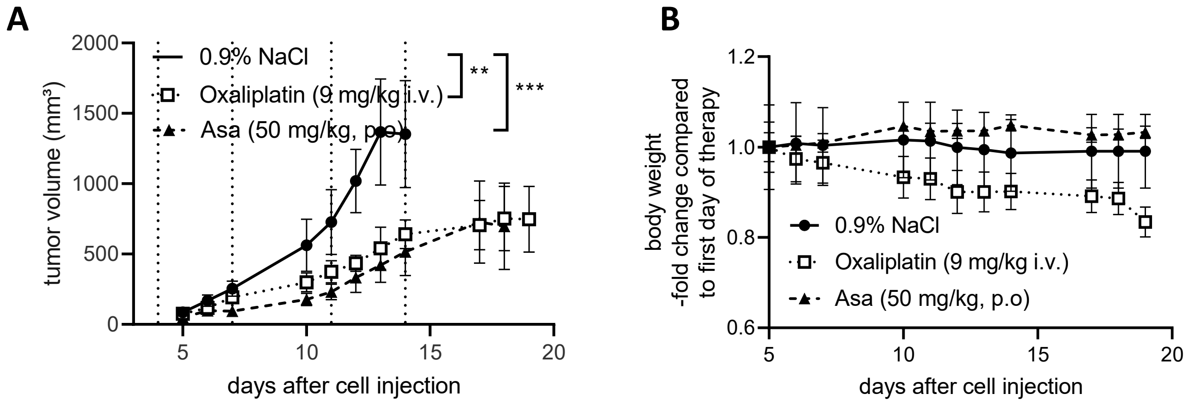

Figure S5: Anticancer activity of **oxaliplatin** and **Asa** in vivo. CT26 -bearing BALB/c mice were treated twice a week for two weeks with a concentration of 9mg/kg (i.v.) for oxaliplatin and 50 mg/kg (p.o.) for Asa. (A) Impact on tumor growth; data are presented as means ± SEM. Dashed lines indicate treatments days. Statistical significance was calculated by two-way ANOVA and Tukey's multiple comparison test (**p < 0.01, ***p < 0.001). (B) Impact on body weight over the treatment period.

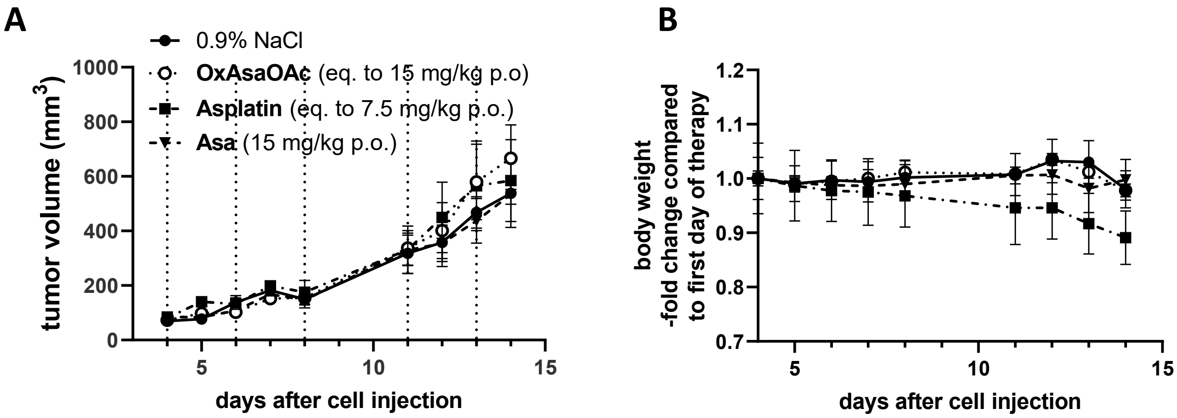

Figure S6: Oral anticancer activity of **OxAsaOAc, asplatin** and **Asa** in vivo. CT26 -bearing BALB/c mice were treated twice a week for two weeks with a concentration equimolar to of 15 mg/kg Asa in case of OxAsaOAc and 7.5 mg/kg in case of asplatin. (A) Impact on tumor growth; data are presented as means ± SEM. Dashed lines indicate treatments days. (B) Impact on body weight over the treatment period.

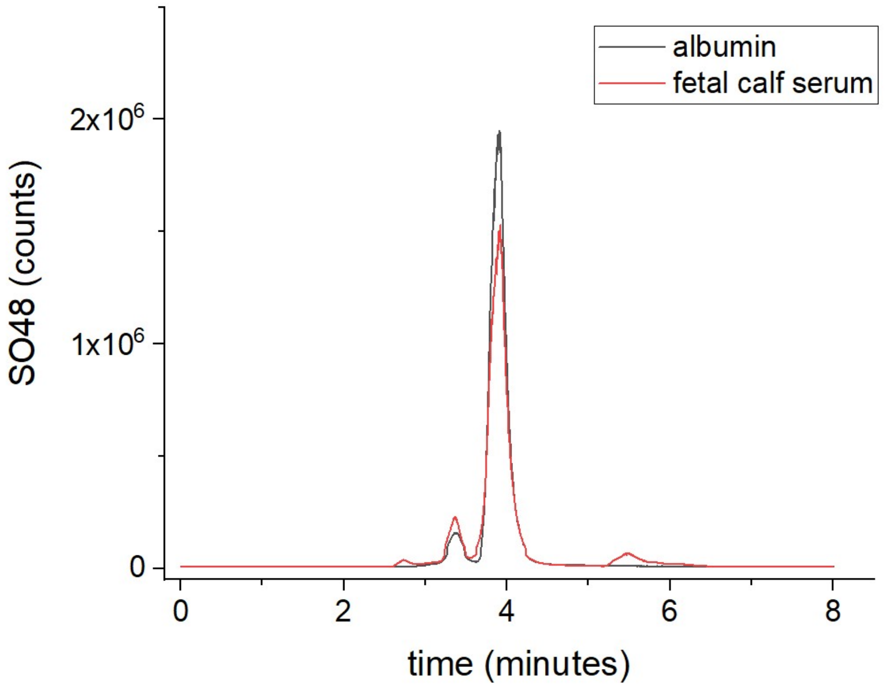

Figure S7: Sulfur traces of fetal calf serum (+150 mM phosphate buffer, pH 7.4) as well as pure albumin in phosphate buffer (50 mM, pH 7.4), both measured by SEC-ICP-MS. The small peak at ~3.3 min corresponds to the albumin dimer.

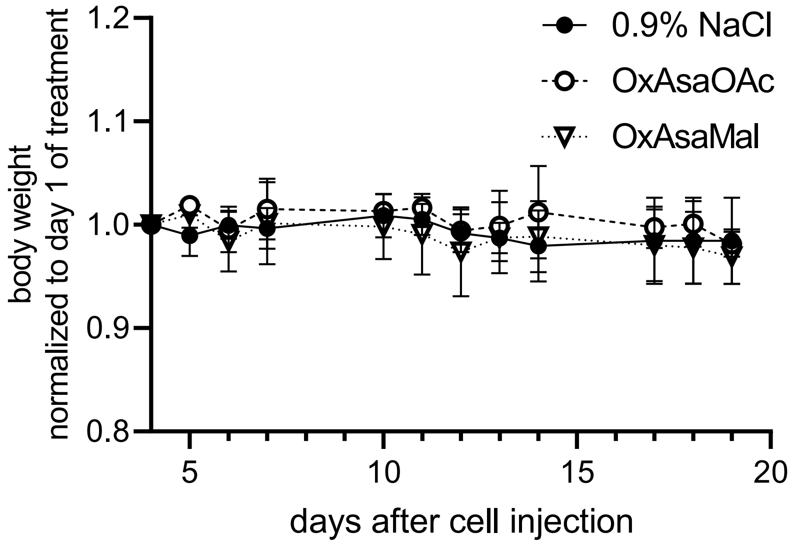

Figure S8: Impact on body weight over the treatment period of OxAsaOAc and OxAsaMal (i.v.) in immune-competent Balb/c mice. Mice were treated equimolar to 9 mg/kg oxaliplatin.

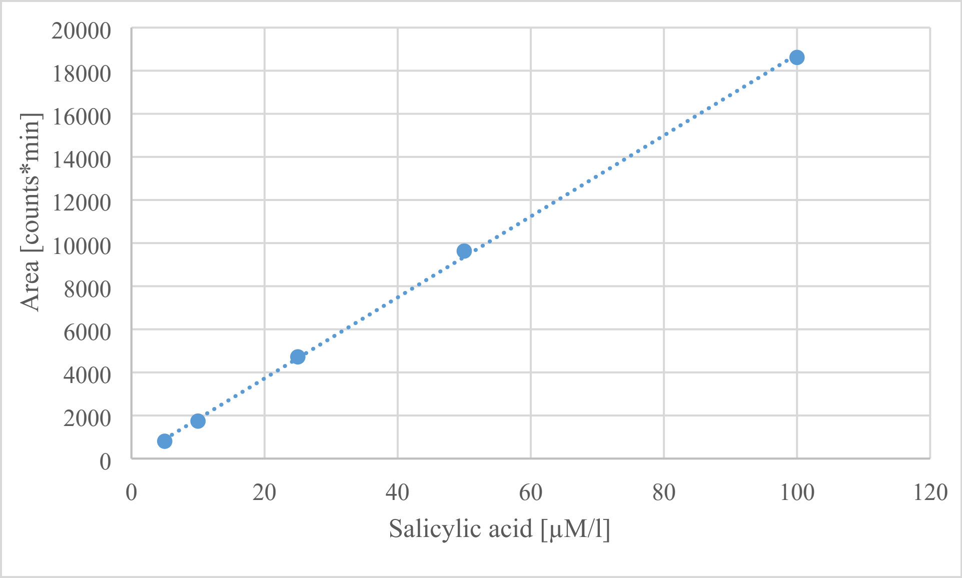

Figure S9: Calibration for the extraction of salicylic acid out of biological media.

RF power	1550
Nebulizer	MicroMist
Spray chamber	Scott double-pass
Monitored isotopes	^{185}Re, ^{195}Pt, ^{196}Pt
Plasma gas	15 l/min
Nebulizer gas	1.08 l/min
Auxiliary gas	0.90 l/min
Cone material	Ni
Integration time	0.1 s
Number of replicates	10
Number of sweeps	100

Table S1: ICP-MS parameters for the measurement of platinum levels in cells, tumor- and organ-tissue.

HPLC column:	Acquity UPLC BEH 200Å 1.7 µm, 4.6x150 mm
Eluent:	50 mM CH_3COONH_4, pH = 6.8
Flow rate:	400 µL/min
Injection volume:	5 µL
Column temperature:	37°C
Autosampler temperature:	37°C

Table S2: SEC-HPLC parameters for SEC-ICP-MS measurements.

Nebulizer:	Quartz
Spray chamber:	Scott type
Nebulizer gas flow:	1.08 L/min
Aux. gas flow:	0.9 L/min
Plasma gas flow:	15 L/min
Reaction gas (oxygen):	30 %
ICP RF power:	1550 W
m/z measured:	195, 48

Table S3: ICP-MS parameters for SEC-ICP-MS measurements.
